# Leishmanicidal Activity of (+)-Phyllanthidine and the Phytochemical Profile of *Margaritaria nobilis* (Phyllanthaceae)

**DOI:** 10.3390/molecules201219829

**Published:** 2015-12-11

**Authors:** Lienne S. Moraes, Marcio R. H. Donza, Ana Paula D. Rodrigues, Bruno J. M. Silva, Davi S. B. Brasil, Maria das Graças B. Zoghbi, Eloísa H. A. Andrade, Giselle M. S. P. Guilhon, Edilene O. Silva

**Affiliations:** 1 Laboratório de Parasitologia e Biologia Estrutural, Instituto de Ciências Biológicas, Universidade Federal do Pará, Belém, Pará 66075-110, Brazil; liennemoraes@hotmail.com (L.S.M.); bjmartins@ufpa.br (B.J.M.S.); edilene@ufpa.br (E.O.S.); 2 Instituto Nacional de Biologia Estrutural e Bioimagem, Universidade Federal do Rio de Janeiro, Rio de Janeiro 21941-901, Brazil; anarodrigues@iec.pa.gov.br; 3 Programa de Pós-Graduação em Química, Instituto de Ciências Exatas e Naturais, Universidade Federal do Pará, Belém, Pará 66075-110, Brazil; marciodonza@hotmail.com (M.R.H.D.); davibb@ufpa.br (D.S.B.B.); eloisandrade@ufpa.br (E.H.A.A.); 4 Laboratório de Microscopia Eletrônica, Instituto Evandro Chagas, Secretaria de Vigilância em Saúde, Ministério da Saúde, Belém, Pará 66090-000, Brazil; 5 Coordenação de Botânica, Museu Paraense Emílio Goeldi, Belém, Pará 66077-901, Brazil; gracazoghbi@gmail.com

**Keywords:** *Leishmania (L.) amazonensis*, leishmanicidal activity, alkaloid (+)-phyllanthidine

## Abstract

The effects of the Securinega alkaloid (+)-phyllanthidine on *Leishmania (L.) amazonensis* and the first chemical investigation of *Margaritaria nobilis* L.f. (Phyllanthaceae) are described. Treating the parasites with this alkaloid caused a dose-dependent reduction in promastigote growth of 67.68% (IC_50_ 82.37 μg/mL or 353 µM) and in amastigote growth of 83.96% (IC_50_ 49.11 μg/mL or 210 µM), together with ultrastructural alterations in the promastigotes. No cytotoxic effect was detected in mammalian cells (CC_50_ 1727.48 µg/mL or CC_50_ 5268 µM). Classical chromatographic techniques and spectral methods led to the isolation and identification of betulinic acid, kaempferol, corilagin, gallic acid and its methyl ester, besides (+)-phyllanthidine from *M. nobilis* leaves and stems. *Margaritaria nobilis* is another source of the small group of Securinega alkaloids, together with other Phyllanthaceae (Euphorbiaceae s.l.) species. The low toxicity to macrophages and the effects against promastigotes and amastigotes are suggestive that (+)-phyllanthidine could be a promising antileishmanial agent for treating cutaneous leishmaniasis.

## 1. Introduction

Leishmaniasis is an anthropozoonotic and neglected disease, and it is considered a major health problem worldwide [1]. This disease is caused by parasites belonging to the genus *Leishmania*, and it affects approximately 12 million people worldwide [2,3,4]. It is an endemic disease in 98 countries, with most cases reported in tropical and subtropical countries [5]. Leishmaniasis is transmitted by hematophagous phlebotomine sand flies [2]. *Leishmania (L.) amazonensis* can cause diffuse cutaneous leishmaniasis or anergic diffuse cutaneous leishmaniasis (ADCL). At present, the primary drugs used for leishmaniasis are Glucantime^®^, Pentostan and Amphotericin B [6,7]; however, these drugs are highly toxic and administered in an invasive manner, requiring long treatment and promoting several adverse side effects [8,9]. The search for new products for leishmaniasis treatments is the current target of many studies.

Natural products are a potential source of new agents for the treatment of many diseases, including leishmaniasis. Singh and coworkers listed over 200 plant products that have shown antileishmanial properties and, among these, almost 100 compounds are alkaloids, including quinoline, indole, naftyl-, benzyl- and isoquinoline, steroidal and diterpene alkaloids, benzoisoquinolizidine and pyrimidine-β-carboline alkaloids [10].

*Margaritaria* *nobilis* L.f. is included in the Phyllanthaceae, a morphologically diverse pantropical family of approximately 2000 species and 60 genera that was segregated from Euphorbiaceae s.l. along with Pandaceae, Picrodendraceae and Putranjivaceae [11]. This species is a shrub and it is widely distributed in Brazil, through the Amazon, Atlantic Forest, “Cerrado” and “Pantanal” [12].

**Figure 1:**
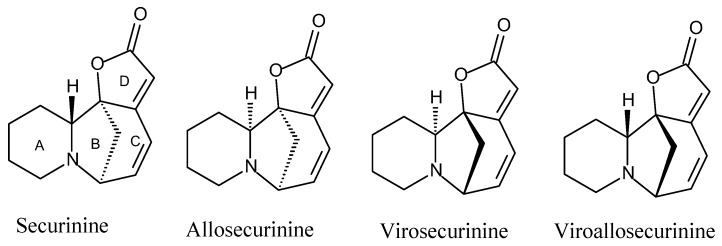
Structures of some Securinega alkaloids.

Chemical studies of *Margaritaria* species have led to the isolation of several Securinega alkaloids, including securinine, allosecurinine, phyllanthine, epiphyllanthine, phyllochrysine, securinol, viroallosecurinine, (+)-phyllanthidine and dihydroallosecurinine from *M. indica* [13,14] and *M. discoidea* [15,16,17]. Securinega alkaloids (or securinane-type alkaloids) are a class of natural products found in a small number of Phyllanthaceae (Euphorbiaceae s.l.) species (*Securinega*, *Phyllanthus*, *Margaritaria*, *Breynia* and *Flueggea*) [18,19]. These alkaloids (Figure 1) exhibit a tetracyclic structure formed by a piperidine or a pyrrolidine (ring A), a 6-azabicycle [3.2.1]-octane (ring B and C) and an α,β-butenolide (ring D), although there are occasional structural differences in the framework, such as in phyllanthidine, in which rings A and C are connected through an oxygen bridge. Securinine is the most common Securinega alkaloid, and it exhibits important biological activities, such as stimulating the Central Nervous System (CNS) and antitumor, anti-malarial and antibacterial activities [19,20]. This group of alkaloids and their derivatives have attracted the chemist interest in total synthesis, as these compounds may serve as potent clinical drugs [18,19,20].

*Margaritaria discoidea* is a well-known medicinal plant in Africa used for the treatment of various diseases [21]. The extracts of this species have been extensively studied and several biological activities were described, including cytotoxic effects on human ovarian cancer cells [21]; weak to moderate antibacterial, antifungal, and anti-HIV activities; and inhibition effects on the growth of *Trypanosoma brucei brucei* and *T. cruzi*, but no effect against *Plasmodium falciparum* and *L. infantum* was observed [17,22]. Moreover, the stem bark of *M. dioscorea* extract suppresses allergy and exhibits anti-inflammatory activity in mice [23] and according to Cho-Ngwa and coworkers [24], the non-polar extracts of this species is a potential source of new microfilaricidal compounds.

To date, there are no chemical studies on *M. nobilis*. The methanol extract of *M. nobilis* bark was inactive against *Aedes aegypt* (LC_100_ > 500 μg/mL), *P. falciparum* (IC_50_ > 10 μg/mL), *L. mexicana* amastigotes (IC_50_ > 10 μg/mL) and *T. cruzi* trypomastigotes (IC_50_ > 10 μg/mL) [25].

Because of the importance of natural products in the search for new structures that exhibit activities against pathogens, especially of alkaloids, the aim of this work was to investigate the chemical composition of *M. nobilis* and the effect of the alkaloid (+)-phyllanthidine on the protozoan *L. (L.) amazonensis*.

## 2. Results and Discussion

### 2.1. Chemical Study

**Figure 2:**
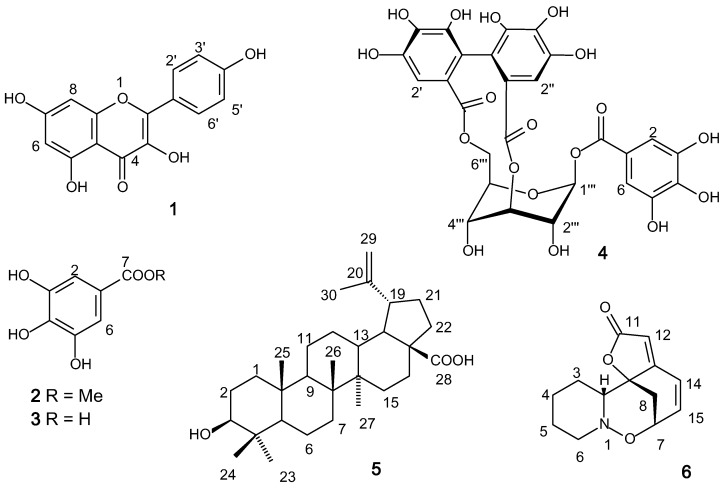
Structures of compounds **1**–**6** isolated from *Margaritaria* *nobilis*.

The chemical study of *M. nobilis* leaves and stems extracts was performed by classic chromatographic techniques and spectrometric methods for the isolation and identification of the substances, respectively. The methanol extract of *M.* *nobilis* leaves yielded the flavonoid kaempferol (**1**), the phenols methyl gallate (**2**) and gallic acid (**3**), and the tannin corilagin (**4**). The methanol extract of the stems led to the isolation of the triterpene betulinic acid (**5**) and the Securinega alkaloid (+)-phyllanthidine (**6**) (Figure 2).

(+)-Phyllanthidine (or *ent*-phyllanthidine) (**6**) was isolated as the major compound of the CH_2_Cl_2_ phase of *M. nobilis* stems. The enantiomer of **6**, (−)-phyllanthidine, was first isolated from *Phyllanthus discoides* [26], although its correct structure (α_D_ − 450) was proposed later after isolation from *Securinega suffruticosa* [27]. Only twenty years after the structural determination of (−)-phyllanthidine, (+)-phyllanthidine (α_D_ + 333) was isolated from *Breynia coronata* [28], and its structure was confirmed by X-ray crystallography. Recently, (+)-phyllanthidine was isolated from *M. discoidea* [17]. The total synthesis of (+)-phyllanthidine (α_D_ + 377) was performed by Carson and Kerr (2006) [29], and no traces of the (−)-isomer were obtained. The α_D_ (+294 ± 4) together with the spectral data and comparison with data from the literature confirmed the identification of **6** as (+)-phyllanthidine. No biological study has been described so far for (+)-phyllanthidine against *L. (L.) amazonensis*.

Compounds **1**–**5** are common plants constituents and have already been tested against *Leshmania* spp. Kaempferol (**1**) showed leishmanicidal activity against *L. (V.) peruviana* and *L. (V) braziliensis* promastigote forms [30]. Methyl galate (**2**) showed a weak antileshmanial activity against *L. mexicana* (IC_50_ 33.6 μM) when compared to miltefosine (IC_50_ 0.5 μM) [25]. Experiments with gallic acid (250 μM) (**3**) induced a modest NO-production on *Leishmania* infected-macrophages but did not reduce parasite viability [31]. Corilagin (**4**) was capable of enhancing the iNOS and cytokine mRNA levels in *L. major*-infected cell when compared with those in non-infected conditions [32]. No antiprotozoal activity against *Leishmania amazonensis* and *L. braziliensis* was detected for betulinic acid (**5**) [33].

### 2.2. Effects of (+)-Phyllanthidine on Host Cell Viability

**Figure 3:**
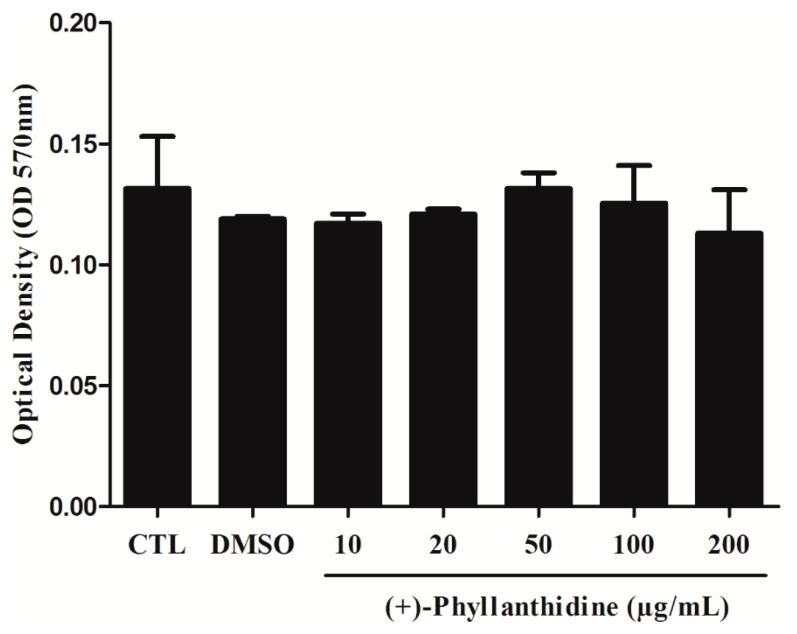
Viability of macrophages treated with different concentrations of (+)-phyllanthidine, when measured by MTT reduction assay after 48 h of treatment. The viability of untreated cells was 100% and no differences were found in treated cells with different concentrations when compared with the control.

The cytotoxic potential of (+)-phyllanthidine on macrophages was evaluated. Macrophage viability was assessed by MTT (3-(4,5-dimethylthiazol-2-yl)-2,5-diphenyltetrazolium bromide) assay, and treating with different concentrations did not promote a decrease in the cell viability of host cells (CC_50_ 1727.48 µg/mL or CC_50_ 5268 µM) (Figure 3) in comparison with untreated cells. These results indicate that (+)-phyllanthidine has no cytotoxic effects on macrophages.

### 2.3. Effects of (+)-Phyllanthidine on Leishmania (L.) amazonensis Promastigotes and Amastigotes

**Figure 4:**
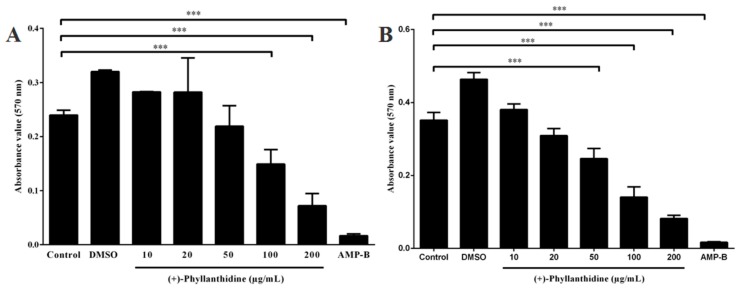
Growth curve of *Leishmania (L.) amazonensis* promastigotes that were treated with different concentrations of (+)-phyllanthidine and 0.5 µg/mL of Amphotericin B (AMP-B): (**A**) treated for 48 h and (**B**) treated for 96 h. *** *p* < 0.001 represents the difference between treated and untreated cells.

**Figure 5:**
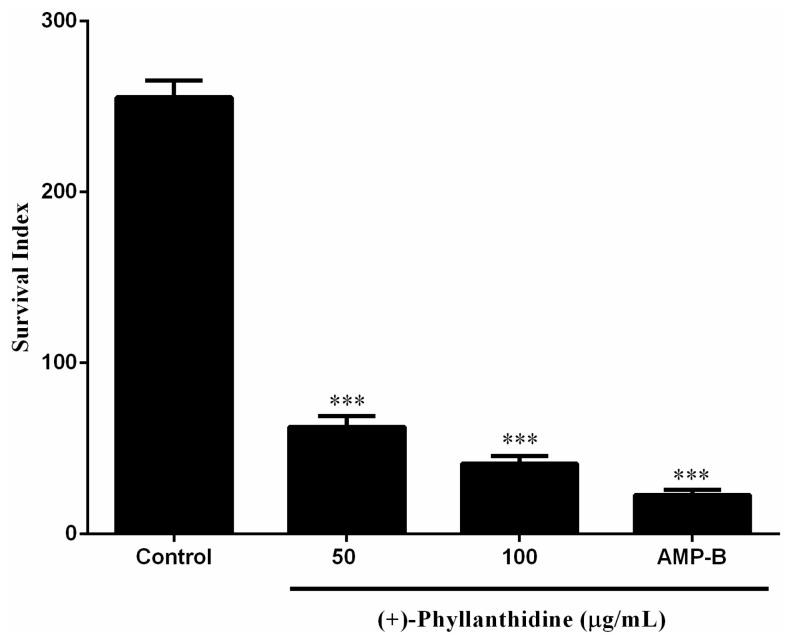
Effect of (+)-phyllanthidine on the amastigote survival for *Leishmania (L.) amazonensis*. Infected murine peritoneal macrophages were treated with 50 and 100 µg/mL of the drug for 48 h after infection. The results are shown in standard derivations of survival inhibition compared with the untreated control. The positive control is Amphotericin B (AMP-B). *** *p* < 0.001 represents the difference between treated and untreated cells.

The actions of leishmanicidal agents and substances used in the leishmaniasis experimental model may act directly on the promastigote and amastigote forms, or they may act indirectly by stimulating the host cell, activating its microbicidal response and inducing *Leishmania* death [34].

(+)-Phyllanthidine was tested to determine its leishmanicidal activity after 96 h of treatment. This alkaloid promoted a dose-dependent effect on *L. (L.) amazonensis* promastigote growth, with an inhibition of 67.68% when treated with 100 µg/mL (Figure 4). The IC_50_ was 82.37 μg/mL (350 µM). Amphotericin B was used as a positive control, and a reduction of 100% was observed in cultures that were treated with 0.5 μg/mL (0.54 µM) for 96 h (Figure 5).

Experiments performed by other researchers [35] with an alkaloid extracted from *Peschiera australis* led to similar results to those of the alkaloid (+)-phyllanthidine, with 90% of growth inhibition of *L. (L.) amazonensis* promastigote after 72 h of treatment. Another study has shown that julocrotine, an alkaloid isolated from *Croton pullei*, exhibited leishmanicidal activity *in vitro* and promoted a 54% reduction in the promastigotes of *L. (L.) amazonensis* after 72 h of treatment [36].

The effects of (+)-phyllanthidine on the amastigote form were also evaluated in *L. (L.) amazonensis*-infected macrophage culture. The results demonstrated that (+)-phyllanthidine significantly inhibited amastigote survival within the macrophages after 48 h of treatment. Concentrations of 50 and 100 μg/mL caused parasite reductions of 75.5% and 83.96%, respectively (IC_50_ 49.11 μg/mL or 210 µM), in comparison with the untreated cells. Amphotericin B caused a reduction of 90.42% after 48 h of treatment with 0.5 µg/mL (0.54 µM) (Figure 5). There was no reduction in the group treated with DMSO (data not shown). These results show that (+)-phyllanthidine has a selective antileishmanial activity with no cytotoxic effects in the mammalian cells showing a selectivity index (SI) of 25.08.

Intracellular amastigotes are responsible for the clinical manifestations of leishmaniasis [37]. Considerable efforts have been done in searching for new substances that act on intracellular forms of *Leishmania* protozoa [38,39,40,41]. Alkaloids and derivatives have shown this effect, such as the diterpene alkaloid derivatives that exhibited antiproliferative action against amastigote forms of *L. infantum* without causing cytotoxic effects in host cells [42]. Moreover, the alkaloid julocrotine caused 80% reduction of *L. (L.) amazonensis* amastigote form, after 72 h when treated with 79 μM [36].

### 2.4. Leishmania Ultrastructural Alterations after (+)-Phyllanthidine Treatment

Electron microscopy is a useful tool to study and determine drug effectiveness as well as changes in parasite morphology, and it can also be used in the discovery of new targets that could help elucidate the mechanism of new therapy actions [43,44].

**Figure 6:**
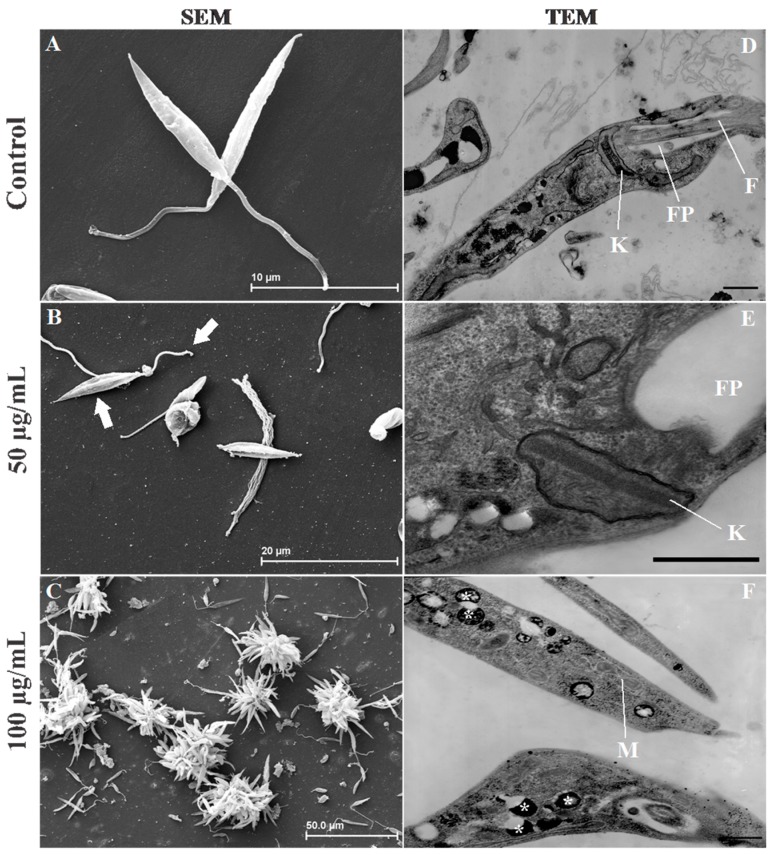
Effect of (+)-phyllanthidine on the ultrastructure of *Leishmania (L.) amazonensis* promastigotes, which were observed by scanning (SEM) (**A**–**C**) and transmission (TEM) (**D**–**F**) electron microscopy (**A**) Control-promastigote form without treatment; (**B**) promastigote treated with 50 µg/mL, showing the septation of the cell body and the shortening of the flagellum (arrows); and (**C**) promastigotes treated with 100 µg/mL; observe the cellular debris and formation of rosettes; (**D**) Control without treatment, showing normal characteristics; (**E**) promastigote forms treated with 50 µg/mL of alkaloid. Note the swelling of the kinetoplast; and (**F**) treatment with 100 µg/mL. The M—Mitochondria (*) acidocalcisomes. Scale bars = 2 µm.

The effects of (+)-phyllanthidine on parasite ultrastructures were analyzed. First, the effects of (+)-phyllanthidine on the *Leishmania* promastigote surface were evaluated by scanning electron microscopy (SEM). The analysis showed the typical morphology of untreated promastigotes (Figure 6A) and alterations in the flagellum and cellular body of promastigote forms after they were treated with the alkaloid. In promastigotes treated for 96 h with 50 µg/mL, the flagella was shortened and body cell septation was observed (Figure 6B). A cluster of cells known as rosettes were noted, the flagella was shortened, the cell body had an atypical morphology and cellular debris was present when 100 µg/mL was used (Figure 6C). In addition, according to the TEM analysis, there were ultrastructural changes in the promastigote of *L.(L.) amazonensis*. Untreated parasites showed typical morphology (Figure 6D), and 96 h of treatment showed significant changes induced by (+)-phyllanthidine. The 50 µg/mL treatment with the alkaloid promoted kinetoplast swelling (Figure 6E).

Some authors have shown changes in *Leishmania* kinetoplasts after using natural products, such as the alkaloid julocrotine [36] and the oil from *Ocimum gratissimum* [45]. This organelle is present only in trypanosomatids, and it is a major target for drug action because of its unique structure and function [44,46].

(+)-Phyllanthidine also caused an increase in the number of acidocalcisome-like structures in promastigotes treated with 100 µg/mL of (+)-phyllanthidine (Figure 6E,F). Guimarães *et al.* (2010) [36] demonstrated similar results, with an increase in the number of acidocalcisome-like structures with treatment with julocrotine.

## 3. Materials and Methods

### 3.1. Chemicals and Instruments

Methanol (MeOH), ethyl acetate (EtOAc), dichloromethane (CH_2_Cl_2_), *n*-butanol (*n*-BuOH) and hexane, HPLC, ACS or PA grade, were purchased from Tedia (Fairfield, OH, USA) , Synth (Diadema, SP, Brazil), Quimex (Cotia, SP, Brazil) or Isofar (Duque de Caxias, RJ, Brazil). Column chromatographic separations were performed on silica gel 70–230 mesh from Vetec (Duque de Caxias, RJ, Brazil) or Merck (Darmstadt, Germany). Fractions were monitored by TLC on silica gel, GF254 from Merck or TLC silica gel from Vetec (Duque de Caxias, RJ, Brazil). NMR spectra were acquired in CDCl_3_, CD_3_OD, pyridine-*d*_5_ or DMSO-*d*_6_ from Tedia (Andover, MA, USA) on a Mercury 300/Varian instrument (^1^H: 300 MHz; ^13^C: 75 MHz, Palo Alto, CA, USA), and chemical shift referencing was performed with internal solvent resonances calibrated to tetramethylsilane (TMS) at 0.00 ppm. The optical rotation was measured with a 341 Perkin Elmer polarimeter (Wellesley, MA, USA) at 589 nm. IV spectrum was recorded on IRPrestige-21 FTIR Shimadzu spectrometer (Kyoto, Japan). The GC-MS data were recorded on a Thermo DSQ GC-MS instrument (Austin, TX, USA) with a DB-5 column. Melting point was determined on a Quimis apparatus (Diadema, SP, Brazil).

### 3.2. Plant Material

Leaves and stems of *M.* *nobilis* were collected in the municipality of Igarapé-açu, State of Pará (PA), Brazil (January 2009). A voucher specimen (MG 191,133) was deposited in the herbarium at the Museu Paraense Emílio Goeldi (MPEG), Belém (PA), Brazil.

### 3.3. Extraction and Partition

The samples were dried for 7 days in an air-conditioned room (at low humidity) and then ground. The air-dried powered leaves (1900 g) and stems (2000 g) were extracted at room temperature with hexane (7 days) and methanol (14 days). The solutions were concentrated under vacuum to yield the hexane extracts (17 g of leaves extract and 18 g of stems extract) and methanol extracts (151 g of leaves extract and 119 g of stems extract). Part of the methanol extracts (20 g of leaves extract and 40 g of stems extract) were solubilized in MeOH-H_2_O (3:1, *v*/*v*) and partitioned with hexane, CH_2_Cl_2_, EtOAc and *n*-BuOH. The solutions were concentrated under reduced pressure to give the leaves phases (hexane phase: 2.38 g, CH_2_Cl_2_ phase: 4.50 g, EtOAc phase: 11.00 g and *n*-BuOH phase: 7.53 g) and stems phases (hexane phase: 2.26 g, CH_2_Cl_2_ phase: 4.00 g, EtOAc phase: 7.06 g and *n*-BuOH phase: 0.37 g).

### 3.4. Isolation and Identification of the Compounds

All identified substances were isolated by column chromatographic (CC) procedures on silica gel by using mixtures of hexane, EtOAc and MeOH in increasing order of polarity as mobile phases. The CC fractioning of the CH_2_Cl_2_ phase of the leaves yielded kaempferol (compound **1**, 46 mg) and methyl gallate (compound **2**, 23 mg) from fractions eluted with hexane–EtOAc 20% and 40%, respectively. Gallic acid (compound **3**, 88 mg) and corilagin (compound **4**, 41 mg) were isolated from the EtOAc phase of the leaves from fractions eluted with hexane–EtOAc 50% and EtOAc 100%, respectively. The fractioning of the CH_2_Cl_2_ phase of the stems yielded betulinic acid (compound **5**, 47 mg) from the fraction eluted with hexane–EtOAc 25%; the fraction eluted with hexane–EtOAc 30% was further purified by CC, and the fractions eluted with hexane-EtOAc 12.5 and 14% yielded (+)-phyllanthidine (compound **6**, 140 mg). The structures of the isolated compounds were proposed from NMR, IV and MS data, through comparison with data from the literature.

### 3.5. Characterization of Compounds ***1***–***6***

*Compound* **1**. Yellow powder; m.p. 273–275 °C; ^1^H-NMR (CD_3_OD, δ_H_): 8.08 (d, *J* = 8.7 Hz, H-2′ and H-6′), 6.90 (d, *J* = 8.7 Hz, H-3′ and H-5′), 6.39 (d, *J* = 2.1 Hz, H-8), 6.18 (d, *J* = 2.1 Hz, H-6); ^13^C-NMR (CD_3_OD, δ_C_): 177.4 (C-4), 165.6 (C-7), 162.5 (C-4′), 160.5 (C-5), 158.3 (C-9), 148.1 (C-2), 137.2 (C-3), 130.7 (C-2′ and C-6′), 123.7 (C-1′), 116.3 (C-3′ and C-5′), 104.5 (C-10), 99.3 (C-6), and 94.4 (C-8). According to these data and those reported in literature [47] and co-TLC with authentic sample, compound **1** was identified as kaempferol.

*Compound* **2**. Colorless, amorphous powder; ^1^H-NMR (CD_3_OD, δ_H_): 7.02 (s, H-2 and H-6) and 3.80 (s, OMe); ^13^C-NMR (CD_3_OD, δ_C_): 169.0 (C-7), 146.5 (C-3 and C-5), 139.8 (C-4), 121.4 (C-1), 110.0 (C-2 and C-6), and 52.3 (OMe). Compound **2** was identified as methyl gallate from comparison with NMR data [48] and co-TLC with authentic sample.

*Compound* **3**. Colorless, amorphous powder; ^1^H-NMR (CD_3_OD, δ_H_): 7.04 (s, H-2 and H-6); ^13^C-NMR (CD_3_OD, δ_C_): 170.4 (C-7), 146.5 (C-3 and C-5), 139.8 (C-4), 122.0 (C-1), and 110.3 (C-2 and C-6). Compound **3** was identified as gallic acid from NMR data [49] and co-TLC with authentic sample.

*Compound* **4**. Colorless, amorphous powder. IV υ_max_ (KBr) cm^−1^: 3373, 1717, 1616, 1508, 1445. The ^1^H- and ^13^C-NMR spectra of **4** showed characteristic signals for a hydrolyzable tannin with galloyl, 1-*O*-galloyl-3,6-(*R*)-hexa-hydroxydiphenoyl (HHDP) and glucose units. ^1^H-NMR (DMSO-*d*_6_, δ_H_): 7.02 (s, H-2 and H-6 galloyl unit), 6.57 (s, H-2′ HHDP unit), 6.50 (s, H-2″ HHDP unit), 6.20 (d, *J* = 7.2 Hz, H-1′′′ glucose unit), 4.60 (ls, H-3′′′ glucose unit), 4.36 (t, *J* = 8.1 H, H-5′′′ glucose unit), 4.27–4.21 (m, H-6a′′′ and H-4′′′ glucose unit), 3.96 (dd, *J* = 10.8 and 8.7 Hz, H-6b′′′ glucose unit), 3.88 (d, *J* = 7.2 Hz, H-2′′′ glucose unit); ^13^C-NMR (DMSO-*d*_6_, δ_C_): 165.1 (C=O galloyl unit), 145.9 (C-3 and C-5, galloyl unit), 139.4 (C-4, galloyl unit), 119.0 (C-1, galloyl unit), 109.3 (C-2 and C-6, galloyl unit), 167.4 and 167.0 (C=O HHDP), 145.1 and 145.0 (C-5′ and C-5″), 144.6 and 144.3 (C-3′ and C-3″), 135.8 and 135.7 (C-4′ and C-4″), 124.1 and 123.4 (C-1′ and C-1″), 116.1 and 115.8 (C-6′ and C-6″), 107.2 and 106.3 (C-2′ and C-2″), 92.5 (C-1′′′, anomeric carbon), 77.8 (C-3′′′), 76.6 (C-5′′′), 71.9 (C-2′′′), 62.4 (C-4′′′), and 64.2 (C-6′′′). Compound **4** was identified as corilagin from spectral data [50].

*Compound* **5**. Colorless powder. IV υ_max_ (KBr) cm^−1^: 3464, 2941, 2868, 1685, 1652, 1450, 1226, 1186. ^1^H-NMR (pyridine-*d*_5_, δ_H_): 3.54 (superimposable signals, H-3 and H-18), 4.96 and 4.79 (both ls, H-29), 0.84, 1.03, 1.08, 1.09, 1.25 (all methyl s, H-25, H-24, H-26, H-27, and H-23), 1.81 (ls, H-30); ^13^C-NMR (pyridine-*d*_5_ δ_C_): 179.1 (C-28), 151.5 (C-20), 110.2 (C-29), 78.3 (C-3), 56.8 (C-17), 56.1 (C-5), 51.2 (C-9), 50.0 (C-18), 48.0 (C-19), 43.1 (C-14), 41.3 (C-8), 39.8 (C-1), 39.5 (C-4), 38.8 (C-13), 37.8 (C-10), 37.7 (C-22), 35.0 (C-7), 33.1 (C-16), 31.4 (C-21), 30.5 (C-15), 28.9 (C-23), 28.5 (C-2), 26.3 (C-12), 21.4 (C-11), 19.7 (C-30), 19.0 (C-6), 16.6 (C-24, C-25, C-26), and 15.1 (C-27). Compound **5** was identified from spectral data as betulinic acid [51,52].

*Compound* **6**. Colorless powder; ${\left[\alpha \right]}_{\text{D}}^{20}$ +296 ± 4 (*c* 0.2 mg/mL, CHCl_3_); ^1^H-NMR (CDCl_3_, δ_H_): 6.86 (d, *J* = 9.3 Hz, H-14), 6.29 (dd, *J* = 9.3 and 5.9 Hz, H-15), 5.83 (s, H-12), 4.71 (ddd, *J* = 5.7, 3.1 and 2.3 Hz, H-7), 3.17 (ld, *J* = 9.6 Hz, H-6b), 2.77 (dd, *J* = 11.3 and 2.6 Hz, H-2), 2.58 (ddd, *J* = 11.0, 11.0 and 9.6 Hz, H-6a), 2.53 (dd, *J* = 11.5 and 3.1 Hz, H-8b), 2.00 (dd, *J* = 11.5 and 2.3 Hz), 1.76 (m, H-3a), 1.70 (m, H-5b), 1.65 (m, H-4a), 1.51 (m, H-5a), 1.22 (m, H-4b), and 0.80 (dddd, *J* = 11.3, 11.3, 11.3 and 3.9 Hz, H-3b). ^13^C-NMR (CDCl_3_, δ_C_): 172.0 (C-11), 164.2 (C-13), 134.3 (C-15), 126.3 (C-14), 113.1(C-12), 82.8 (C-9), 71.1 (C-2), 70.8 (C-7), 56.0 (C-6), 40.5 (C-8), 25.1 (C-5), 23.8 (C-3), and 23.1 (C-4). EI-MS: *m*/*z* 233 (1%), *m*/*z* 100 (100%), and *m*/*z* 83 (8%). According to these data and those reported by Carson and Kerr (2006) [29], compound **6** was identified as (+)-phyllanthidine.

### 3.6. (+)-Phyllanthidine Dilution

The alkaloid (+)-phyllanthidine was solubilized in DMSO (dimethyl sulfoxide) and then diluted in RPMI (Roswell Park Memorial Institute) or DMEM (Dulbecco’s Modified Eagle’s Medium). The stock solution had a concentration of 1 mg/mL, and the concentrations used in the experiments were diluted from this stock.

### 3.7. Leishmania (L.) amazonensis Parasites

Promastigotes from *Leishmania (L.) amazonensis* (MHOM/BR/26361) were obtained in NNN (Neal, Novy and Nicolle) medium from the Instituto Evandro Chagas and subsequently maintained in RPMI medium supplemented with 10% heat-inactivated fetal bovine serum (FBS) at 25 °C.

### 3.8. Peritoneal Macrophage Culture

Macrophages were obtained from the peritoneal cavity of male mouse BALB/c (*Mus musculus*), and the animals were sacrificed in a CO_2_ chamber (Insight^®^). The material was harvested with Hank’s solution, concentrated by centrifugation at 4 °C, cultured in a 24-well plate and incubated in an atmosphere containing 5% CO_2_ at 37 °C for 1 h. After that, non-adherent cells were washed with DMEM and incubated for 24 h with DMEM medium supplemented with 10% FBS. The experimental protocol was approved by the Committee on the Ethics of Animal Experiments (CEPAE, grant number 046-2015) of Universidade Federal do Pará.

### 3.9. Cytotoxicity Assays of Host Cells

MTT is a tetrazolium salt that is converted into blue formazan crystals that are insoluble in water after cleavage by mitochondrial dehydrogenases, which causes them to accumulate in viable cells. The procedure was performed according to Fotakis and Timbrell (2006) [53], with some modifications. Macrophages treated with 10, 20, 50, 100 and 200 µg/mL of (+)-phyllanthidine for 48 h and untreated cells were incubated with MTT (0.5 mg/mL) dissolved in phosphate-buffered saline (PBS), pH 7.2 for 3 h in a humidified atmosphere containing 5% CO_2_ at 37 °C. After that, the macrophages were washed once with PBS pH 7.2, and DMSO was added to the wells. The plate was shaken for 10 min for complete solubilization. The absorbance of each solution was recorded at an optical density (OD) of 570 nm using a spectrophotometer (Bio-Rad Model 450 Microplate Reader). The assay specificity was determined by using non-viable cells treated with 10% formaldehyde in PBS. 10 μL of DMSO was used as the control.

### 3.10. Anti-Promastigote Assay

*Leishmania (L.) amazonensis* promastigotes (10^6^ parasites/mL) were seeded in 24-well plates with RPMI medium without phenol, the medium was supplemented with 10% FBS, and the cells were then treated with different concentrations of (+)-phyllanthidine (10, 20, 50, 100 and 200 µg/mL). The cultures were incubated at 25 °C for 4 days without medium replacement. Every 24 h, aliquots were harvested and promastigotes were incubated with 20 µL of MTT (2 µg/mL) for 4 h. Then, 20 µL of DMSO was added to the wells, and the plate was allowed to shake for 30 min for complete solubilization. One known anti-leishmanial drug (Amphotericin B—0.5 µg/mL) was used as a positive control.

### 3.11. Anti-Amastigote Assay

Adherent peritoneal macrophages were infected with *Leishmania (L.) amazonensis* promastigotes (stationary growth phase) at a parasite/macrophage ratio of 10:1 and incubated for 3 h at 37 °C and 5% CO_2_. After 3 h, free parasites were removed by washing with PBS pH 7.2, and the cultures were treated with 50 and 100 µg/mL (+)-phyllanthidine for 48 h post infection, without replacing the culture medium. The cells were washed with PBS pH 7.2, fixed with methanol and stained with Giemsa. The number of parasites was determined by examining three cover slips for each treatment. At least 100 infected macrophages were counted, and the results were expressed as the survival percentage, in comparison with controls. Amphotericin B (0.5 µg/mL) was used as a positive control. The selectivity index (SI) was calculated as the ratio between the cytotoxicity and antiparasitic activity against intracellular amastigotes.

### 3.12. Ultrastructural Assay

*Scanning Electron Microscopy (SEM)*: Promastigotes were treated with 50 and 100 µg/mL of (+)-phyllanthidine for 4 days, and they were fixed with paraformaldehyde (4%) and glutaraldehyde (2.5%) in cacodylate buffer (0.1 M) for one hour. The cells were post-fixed in osmium tetroxide (1%), dehydrated in graded ethanol, brought to their critical point with CO_2_, coated with gold and analyzed in a Zeiss LEO 1450VP SEM (Göttingen, Germany).

*Transmission Electron Microscopy (TEM)*: Promastigotes treated with 50 and 100 µg/mL (+)-phyllanthidine for 4 days were fixed with glutaraldehyde (2.5%) and paraformaldehyde (4%) in cacodylate buffer (0.1 M) for one hour. The parasites were post-fixed in an osmium tetroxide solution (1%) and ferrocyanide (0.8%), dehydrated in graded acetone and embedded in epoxy resin. Ultrathin sections were obtained, stained with uranyl acetate/lead citrate and examined with a Zeiss 906E TEM (Göttingen, Germany). 

### 3.13. Statistical Analysis

The results were analyzed with a GraphPad Prism 6.0 (GraphPad Software La Jolla, CA, USA), and an analysis of variance (ANOVA) and Student’s *t*-test were performed to compare the data. Tukey’s test was applied when necessary. All experiments were performed in triplicate and were considered statistically significant at *p* < 0.05.

## 4. Conclusions

The present study shows that *Margaritaria nobilis* is another source the Securinega alkaloid, (+)-phyllanthidine and this alkaloid has leishmanicidal activity against promastigote and amastigote forms of *Leishmania (L.) amazonensis* with no cytotoxic effects in the mammalian cells. The results are indicative that (+)-phyllanthidine could be a promising antileishmanial agent for treating cutaneous leishmaniasis.
